# Oxidative balance and survival in osteoporosis: how antioxidant diets and lifestyles reduce mortality risk

**DOI:** 10.3389/fnut.2025.1541661

**Published:** 2025-07-15

**Authors:** Ziyao Ding, Haixu Qi, Wenbo Li, Changchang Chen, Youwei Li, Jun Sun, Maji Sun, Shuo Feng, Feng Yuan

**Affiliations:** ^1^First Clinical Medical College, Xuzhou Medical University, Xuzhou, Jiangsu, China; ^2^Key Laboratory of Bone Tissue Regeneration and Digital Medicine, Xuzhou Medical University, Xuzhou, China; ^3^Department of Orthopedics, The Affiliated Hospital of Xuzhou Medical University, Xuzhou, China

**Keywords:** osteoporosis, oxidative balance score, antioxidant diet, mortality risk, lifestyle factors

## Abstract

**Background:**

Osteoporosis (OP) is a global health issue characterized by reduced bone mineral density (BMD) and an elevated risk of fractures. Oxidative stress is implicated in OP pathogenesis, and antioxidant diets and lifestyles may mitigate these effects. This research aims to investigate the correlation between oxidative balance score (OBS) and all-cause mortality in individuals diagnosed with OP.

**Methods:**

This study is based on data from the National Health and Nutrition Examination Survey (NHANES, 2005–2018), which covers 776 OP patients aged 50 or older. OBS was computed using dietary and lifestyle factors, and divided into two categories: dietary oxidative balance score (DOBS) and lifestyle oxidative balance score (LOBS). Participants were grouped into tertiles based on OBS values.

**Results:**

Kaplan–Meier survival analysis revealed significantly higher survival in the high OBS group compared to the low OBS group (*p* = 0.0032). Consistently, weighted Cox proportional hazards models demonstrated a negative association between OBS and all-cause mortality [HR = 0.96, 95% CI (0.94, 0.99), *p* = 0.0036].

**Conclusion:**

OBS is inversely correlated with all-cause mortality among OP patients, underscoring the critical role of antioxidant-rich diets and lifestyle modifications in OP prevention and treatment strategies. Incorporating OBS into clinical practice may help identify high-risk individuals and guide targeted interventions to reduce mortality risk.

## 1 Introduction

Osteoporosis (OP), as the World Health Organization defined in 1994, is a progressive, systemic skeletal disease characterized by reduced bone mineral density (BMD) and deterioration of bone microarchitecture, which contribute to increased bone fragility and a heightened risk of fractures (1). With the global aging trend, osteoporosis OP has become a major public health issue (2), affecting approximately 200 million people worldwide. One in three women and one in five men over 50 will experience an osteoporotic fracture, leading to significant disability, death, and rising healthcare costs—over $19 billion annually in the U.S. alone, projected to reach $25 billion by 2025 (3). If left untreated, this increase is likely to lead to more cases of recurrent fractures, which can cause disability and even premature death (4). This impact of OP imposes a significant burden on patients and their families, exacerbating healthcare expenditures associated with OP and imposing a considerable socioeconomic strain, thereby highlighting its status as a major global public health issue (3).

Oxidative stress refers to an imbalance between oxidative and antioxidant systems, resulting in a relative excess of reactive oxygen species (ROS), which can potentially lead to cell and tissue damage and death (5). The critical involvement of ROS in the initiation and progression of various pathological conditions is widely acknowledged (6). Recent studies have increasingly demonstrated the significant contribution of oxidative stress to the development and progression of OP among these factors (7, 8). One of the central mechanisms underlying this process is the decoupling of the functional balance between osteoblasts and osteoclasts induced by ROS which is a crucial reason for decreased bone mass and deterioration in bone health (9).

In daily life, various factors influence oxidative stress, including antioxidant diets and lifestyles, such as adequate intake of antioxidant nutrients and regular exercise. In contrast, certain lifestyles characterized by pro-oxidative factors, including alcohol use, smoking, obesity, and lack of physical activity, can increase oxidative stress (10). A single factor cannot comprehensively assess oxidative status; therefore, it is recommended to utilize the oxidative balance score (OBS) as a comprehensive indicator for evaluating the equilibrium between pro-oxidants and antioxidants in an individual’s diet and lifestyle. Comprising the dietary oxidative balance score (DOBS) and the lifestyle oxidative balance score (LOBS), OBS enables a more comprehensive assessment of oxidative status (11).

Existing research has confirmed a correlation between OBS and BMD in adults, indicating that diets rich in antioxidants and healthy lifestyles may contribute to a positive impact on bone density (12). However, no study has systematically explored the link between OBS and all-cause mortality, specifically among elderly individuals diagnosed with OP. Given the quantity of severe adverse effects of OP on patient health and public health, this study investigates this correlation to provide insight into the effects of oxidative stress on OP outcomes.

## 2 Materials and methods

### 2.1 Data source

The information utilized in this study was collected from the National Health and Nutrition Examination Survey (NHANES), a comprehensive dataset managed by the National Center for Health Statistics (NCHS) under the U.S. Centers for Disease Control and Prevention (CDC) (12). As a nationwide and representative research, NHANES accumulates nutritional and health data from civilian and non-institutionalized civilians in the United States (13). Each participant provided informed consent before the survey, moreover, the NHANES database excludes any personally identifiable information.

### 2.2 Study population

**Figure 1:**
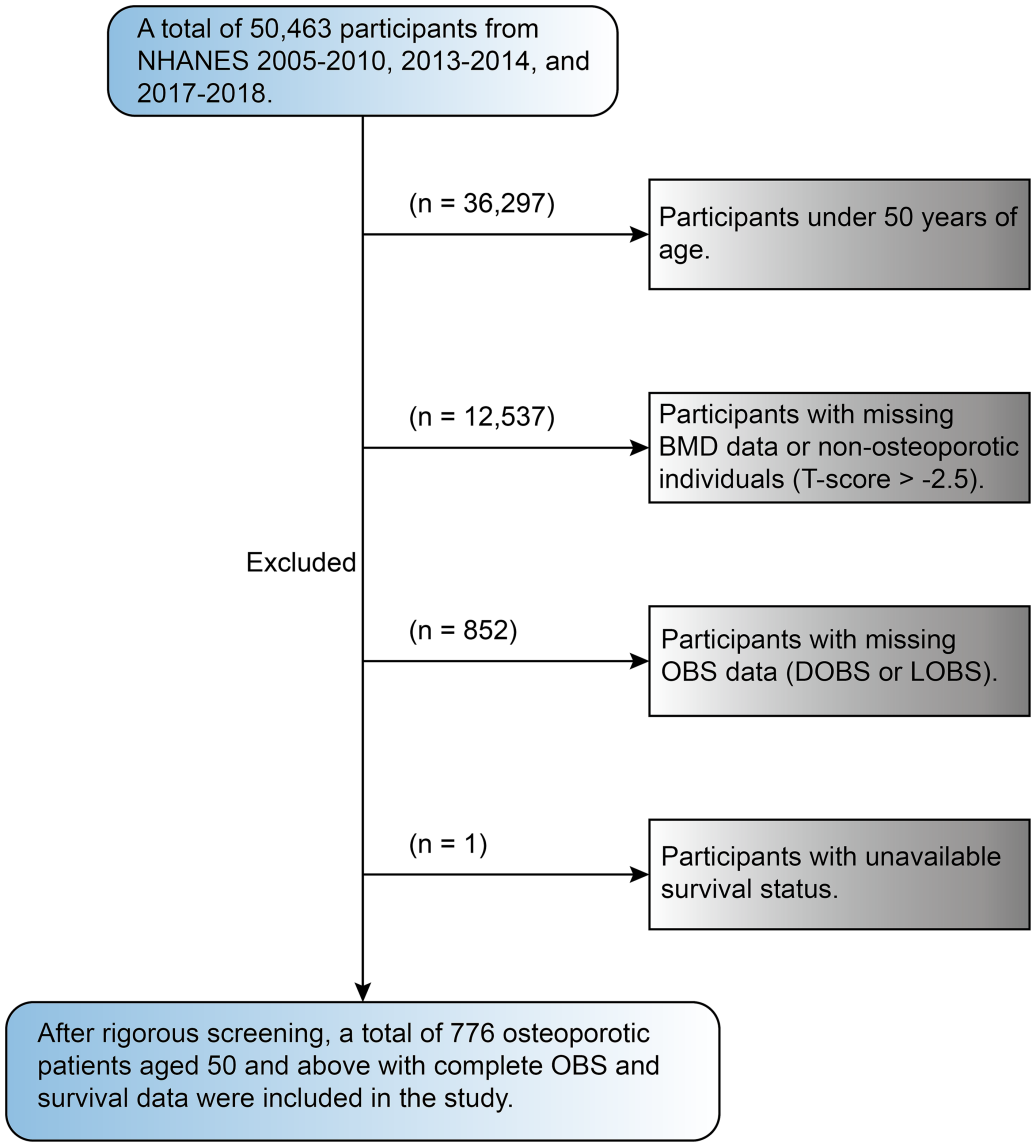
The flow chart of participants in the current study.

This study includes data from survey cycles between 2005 to 2010, 2013 to 2014, and 2017 to 2018 with a total sample size of 50,463. These cycles were specifically chosen by us because they contain complete BMD data for people aged 50 years and older, ensuring a focus on a population relevant to the study’s objectives. The dataset included interview records, laboratory results, blood test data, and other components, which were collected through standardized NHANES protocols to ensure comprehensive and high-quality data acquisition. Exclusion criteria were applied as follows: people under the age of 50 (36,297), as well as those with missing BMD dataset or without OP (12,573), participants missing OBS data (including DOBS and LOBS, 852), and those without available survival status (1). Following the application of rigorous exclusion criteria, the study ultimately included a final cohort of 776 participants (see Figure 1).

### 2.3 BMD measurement and OP definition

The BMD measurements were conducted using advanced dual-energy X-ray absorptiometry (DXA) equipment (Hologic Discovery A) and bone densitometers (Hologic, Bedford, Massachusetts) at Mobile Examination Centers (MECs) by NHANES-certified radiology technicians to ensure accuracy and consistency. The femoral neck and lumbar spine were selected as primary measurement sites, with lumbar spine BMD calculated as the mean of L1–L4. For further analysis, the BMD measurements for the femoral neck and lumbar spine were subsequently transformed into *T*-scores utilizing the subsequent equation: *T*-score = (Participant’s BMD − Reference population mean BMD)/Reference population BMD standard deviation. The study population comprised individuals aged 20–29, both males and females of non-Hispanic White ethnicity, between the years 2005 and 2008, during which BMD measurements were conducted for individuals under 50. Mean values and standard deviations were calculated separately for each sex (14). The diagnosis criterion for OP was a *T*-score ≤ −2.5 at either the lumbar spine or femoral neck. The femoral neck and lumbar spine were the focus of Supplementary Table S1, which provided average values and standard deviations as a reference for the population (15, 16).

### 2.4 Exposure factor: OBS assessment

The OBS comprises both DOBS and LOBS, where DOBS includes 16 dietary nutrients and LOBS includes four lifestyle factors. The OBS encompasses a total of 15 antioxidant components and five pro-oxidant components (17). Nutritional data for the 16 dietary nutrients were derived from two 24-h dietary recalls, averaging components such as dietary carotenoids (retinol equivalent, RE), fiber, riboflavin, and niacin (18). The study examines four primary lifestyle factors: physical activity, body mass index (BMI), smoking, and alcohol consumption; data on physical activity were collected using NHANES’s comprehensive physical activity questionnaire. The metabolic equivalent of task (MET) score was determined by evaluating the weekly duration of physical activity in accordance with the guidelines provided by NHANES. Smoking level was measured by serum cotinine level. Additionally, five pro-oxidant components are identified—BMI, total fat, alcohol consumption, iron, and cotinine, whereas all other components are categorized as antioxidants.

Alcohol consumption was categorized into three groups following criteria established in prior literature: non-drinkers (score 2), light-to-moderate drinkers (score 1: 0–15 g/day for women, 0–30 g/day for men), and heavy drinkers (score 0: ≥15 g/day for women, ≥30 g/day for men), with scores of 2, 1, and 0, respectively. For other components, scoring was determined using sex-specific tertiles. Antioxidant components were assigned scores ranging from 0 to 2 based on their distribution in the lowest to highest tertiles. Conversely, pro-oxidant components were scored in reverse, where individuals in the highest tertile were assigned a score of 0 for pro-oxidant components, while those in the lowest tertile were awarded 2 points. The overall oxidative balance score (OBS) was derived by adding the individual scores of all components, with values spanning from 3 to 36. Elevated OBS values represent greater antioxidant exposure and potentially a lower level of oxidative stress. Detailed scoring criteria for each OBS component are presented in Supplementary Table S2.

### 2.5 Outcome factor: mortality follow-up and confirmation

The determination of mortality status was achieved through the linkage of NHANES data with records from the National Death Index (NDI). The follow-up period for each participant commenced with their NHANES examination. It extended until the time of their demise (if deceased) or December 31, 2019 (if still alive) (19). The primary cause of death was classified using the International Classification of Diseases, Tenth Revision (ICD-10).

### 2.6 Covariate selection

Covariates were selected based on clinical expertise and common confounders included in similar studies (20, 21). Participant characteristics included gender, age group, race family PIR, BMI, education level serum calcium, serum phosphorus, 25-OHD as well as history of hip fracture, history of vertebral fracture, OP treatment, hypertension, hyperlipidemia, cardiovascular disease (CVD), thyroid disease, diabetes, cancer, liver disease and kidney failure. The categories of race included non-Hispanic White, non-Hispanic Black, Mexican American, and other races. There were three levels of education: “below high school,” “High school or equivalent,” and “College or higher.” History of hip fracture and vertebral fracture were determined based on questionnaire responses. OP treatment information was also obtained from questionnaires, which asked participants whether they had ever received OP treatment. The definition of hypertension history was established based on multiple criteria to ensure comprehensive identification, encompassing individuals with a systolic blood pressure measurement equal to or exceeding 140 mmHg, and/or a diastolic blood pressure measurement equal to or surpassing 90 mmHg, and a physician’s documented diagnosis, or the current usage of antihypertensive medication (20). Hyperlipidemia was characterized by a serum total cholesterol level equal to or greater than 6.2 mmol/L, a physician’s clinical diagnosis, or a recommendation to take lipid-lowering medication (21). Cardiovascular diseases (CVD) encompassed conditions such as angina, coronary artery disease, stroke, myocardial infarction, and congestive heart failure, with details collected via questionnaire. Diabetes was diagnosed through a clinical diagnosis by a physician, based on fasting blood glucose levels of at least 7.0 mmol/L or an HbA1c value of 6.5% or higher, as well as the current usage of insulin or other antidiabetic treatments (22). Information on history of thyroid disease, cancer, kidney failure, and liver disease was similarly gathered from participant-provided questionnaires.

### 2.7 Statistical analysis

This analysis accounted for the intricate sampling design of NHANES, incorporating stratification, clustering, and sample weights. Osteoporotic participants were divided into tertiles based on OBS values: low, medium, and high. For baseline analyses, categorical variables were compared utilizing survey-weighted chi-square tests, while continuous variables were examined through survey-weighted linear regression to explore their baseline characteristics. In the survival analysis, Kaplan–Meier survival analysis was conducted to evaluate survival probabilities according to diverse OBS tertiles in the OP population, with log-rank tests assessing significance. A survey-weighted Cox proportional hazards model was utilized to investigate the independent correlation between OBS and all-cause mortality in osteoporotic patients. Three models were constructed for this analysis: Model 1 (single-factor regression model), Model 2 (adjusted for key demographic variables such as gender, race, age, and group), and Model 3 (included comprehensive adjustments by incorporating additional factors such as family PIR, BMI, phosphorus levels and serum calcium, education level, 25-OHD levels), and medical histories of various conditions. These conditions include hip and vertebral fractures, osteoporosis treatment, hypertension, hyperlipidemia, cardiovascular disease, diabetes, thyroid and liver disease, cancer, and renal failure. To explore the possibility of a nonlinear correlation between OBS and all-cause mortality, survey-weighted restricted cubic spline (RCS) curves were applied by us, testing knot positions ranging from three to seven. The model selection process prioritized the Akaike information criterion (AIC), ultimately leading to the adoption of the model with the lowest AIC value, using three parts in the final configuration. To deepen the understanding of the relationships between OBS and mortality, stratified and interaction analyses were conducted, incorporating a broad spectrum of variables such as gender, age, education level, race, history of hip and vertebral fracture, history of OP treatment, history of hypertension, history of hyperlipidemia, history of CVD, history of diabetes, history of thyroid disease, history of liver disease, history of cancer, and history of kidney failure. In order to ensure the robustness of the research conclusions, we conducted sensitivity analyses by excluding participants who died within 2 years of follow-up, mitigating the risk of reverse causation. Missing data were handled through imputation using the k-nearest neighbor (kNN) function from the VIM package (23), with all covariates having less than 20% missing values and missingness assumed to be at random. Statistical analyses were conducted using R software (version 4.3.1) along with EmpowerStats, both recognized for their robustness in analyzing complex datasets. A conclusion testing with a two-sided *p*-value of less than 0.05 was considered statistically significant.

## 3 Results

### 3.1 Baseline characteristics of study participants

**Figure 2:**
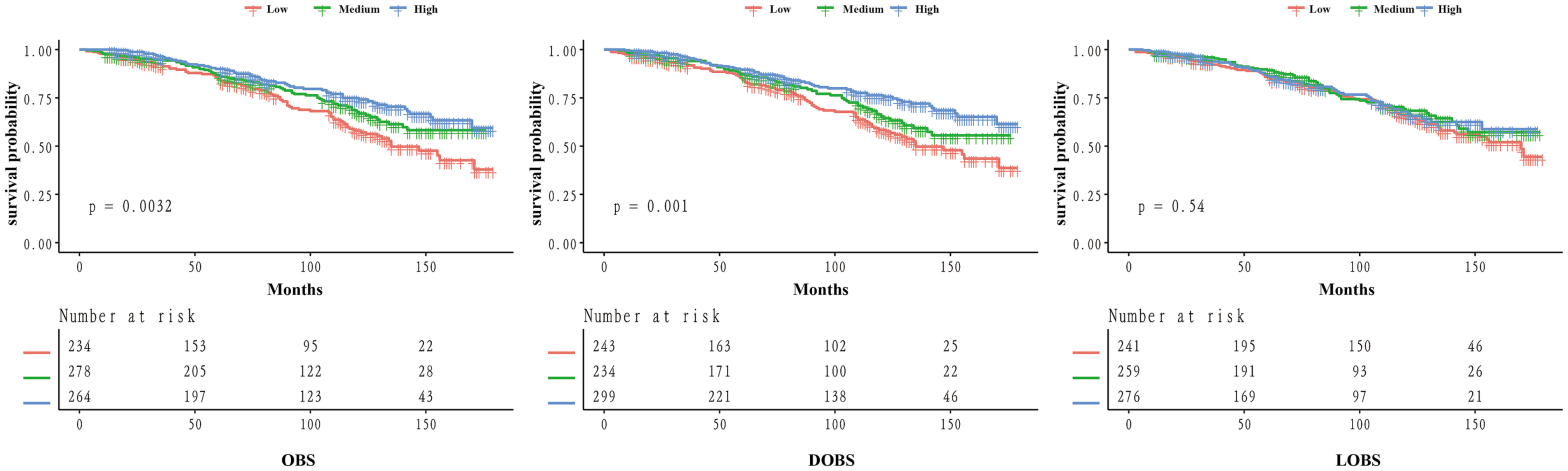
Kaplan–Meier curves of the survival rate for low, medium, and high groups of OBS/DOBS/LOBS.

This study included 776 OP patients aged 50 years and above. Participants were divided into tertiles based on OBS values: low (5, 17), *N* = 243; medium (17, 23), *N* = 234; high (23, 36), *N* = 299 (see Figure 2). Compared to the low OBS group, individuals in the high OBS group exhibited statistically significant differences in several characteristics (*p* < 0.05): they generally exhibited lower BMI, lower likelihood of having a history of hypertension, higher education levels, higher household income, and lower prevalence of CVD, cancer, and kidney failure, as well as lower mortality rates (see Table 1).

**Table 1:** The baseline characteristics by tertiles of the OBS (weighted).

Characteristics	Oxidative balance score (tertile)				*p*-value
	Total, *N* = 776	Low (5, 17), *N* = 243	Medium (17, 23), *N* = 234	High (23, 36), *N* = 299	
Age group, %					0.4763
50–79	84.85 (81.15, 87.94)	81.63 (73.38, 87.75)	86.82 (80.52, 91.30)	85.60 (79.22, 90.26)	
80+	15.15 (12.06, 18.85)	18.37 (12.25, 26.62)	13.18 (8.70, 19.48)	14.40 (9.74, 20.78)	
Gender, %					0.2951
Male	25.76 (21.53, 30.51)	24.62 (16.45, 35.14)	31.81 (22.91, 42.27)	22.20 (15.63, 30.53)	
Female	74.24 (69.49, 78.47)	75.38 (64.86, 83.55)	68.19 (57.73, 77.09)	77.80 (69.47, 84.37)	
Race, %					0.3344
Mexican American	3.50 (2.44, 4.98)	4.77 (2.79, 8.05)	3.68 (1.87, 7.14)	2.51 (1.56, 4.02)	
Non-Hispanic White	83.03 (79.59, 85.99)	79.78 (73.47, 84.90)	83.89 (78.27, 88.26)	84.58 (79.39, 88.66)	
Non-Hispanic Black	3.04 (2.10, 4.37)	4.63 (2.71, 7.80)	2.81 (1.33, 5.85)	2.13 (1.23, 3.69)	
Other races	10.44 (8.09, 13.37)	10.82 (7.00, 16.36)	9.62 (6.88, 13.30)	10.77 (7.28, 15.65)	
Education level, %					<0.0001
Less than high school	17.15 (14.10, 20.70)	29.49 (21.84, 38.50)	17.61 (12.74, 23.83)	8.57 (6.30, 11.55)	
High school or equivalent	26.30 (22.02, 31.08)	38.52 (29.16, 48.81)	23.90 (17.68, 31.46)	19.86 (13.46, 28.30)	
College or above	56.55 (51.50, 61.47)	31.99 (24.75, 40.22)	58.49 (49.83, 66.67)	71.58 (63.74, 78.30)	
Family PIR	2.97 (2.80, 3.15)	2.57 (2.32, 2.81)	3.16 (2.84, 3.49)	3.11 (2.87, 3.35)	0.0023
BMI, kg/cm^2^	25.28 (24.74, 25.82)	26.37 (25.67, 27.07)	25.76 (24.33, 27.18)	24.21 (23.61, 24.81)	0.0001
Calcium, mg/dL	9.44 (9.40, 9.48)	9.42 (9.35, 9.48)	9.41 (9.34, 9.49)	9.47 (9.41, 9.54)	0.4442
Phosphorus, mg/dL	3.84 (3.78, 3.89)	3.77 (3.67, 3.87)	3.83 (3.73, 3.93)	3.89 (3.81, 3.97)	0.2077
25-OHD, nmol/L	79.54 (74.76, 84.31)	78.25 (66.87, 89.62)	79.56 (73.93, 85.18)	80.38 (75.89, 84.88)	0.9266
Lumber *T*-score	−2.35 (−2.47, −2.23)	−2.45 (−2.62, −2.27)	−2.20 (−2.48, −1.91)	−2.40 (−2.59, −2.21)	0.4086
Femoral neck *T*-score	−2.62 (−2.73, −2.52)	−2.69 (−2.82, −2.56)	−2.66 (−2.92, −2.41)	−2.56 (−2.64, −2.48)	0.2630
Hip fracture history, %					0.9096
No	96.55 (94.87, 97.69)	96.82 (91.89, 98.80)	96.90 (92.12, 98.82)	96.10 (92.72, 97.95)	
Yes	3.45 (2.31, 5.13)	3.18 (1.20, 8.11)	3.10 (1.18, 7.88)	3.90 (2.05, 7.28)	
Spine fracture history, %					0.5025
No	97.05 (95.06, 98.25)	95.64 (90.47, 98.06)	97.77 (94.55, 99.11)	97.47 (93.30, 99.07)	
Yes	2.95 (1.75, 4.94)	4.36 (1.94, 9.53)	2.23 (0.89, 5.45)	2.53 (0.93, 6.70)	
OP treatment history, %					0.1414
No	72.01 (66.15, 77.21)	78.36 (68.39, 85.84)	63.97 (50.58, 75.49)	73.52 (64.75, 80.76)	
Yes	27.99 (22.79, 33.85)	21.64 (14.16, 31.61)	36.03 (24.51, 49.42)	26.48 (19.24, 35.25)	
Hypertension history, %					0.0301
No	42.61 (37.45, 47.94)	34.24 (24.49, 45.54)	38.82 (30.19, 48.21)	50.93 (43.17, 58.64)	
Yes	57.39 (52.06, 62.55)	65.76 (54.46, 75.51)	61.18 (51.79, 69.81)	49.07 (41.36, 56.83)	
Dyslipidemia history, %					0.8599
No	45.29 (40.90, 49.76)	43.17 (31.94, 55.14)	44.85 (35.65, 54.42)	47.02 (39.77, 54.41)	
Yes	54.71 (50.24, 59.10)	56.83 (44.86, 68.06)	55.15 (45.58, 64.35)	52.98 (45.59, 60.23)	
CVD history, %					0.0041
No	85.08 (80.60, 88.67)	76.22 (67.74, 83.03)	85.81 (75.76, 92.12)	90.47 (86.12, 93.56)	
Yes	14.92 (11.33, 19.40)	23.78 (16.97, 32.26)	14.19 (7.88, 24.24)	9.53 (6.44, 13.88)	
Diabetes history, %					0.0687
No	88.01 (84.01, 91.12)	85.06 (78.06, 90.11)	84.91 (76.13, 90.85)	92.20 (87.12, 95.38)	
Yes	11.99 (8.88, 15.99)	14.94 (9.89, 21.94)	15.09 (9.15, 23.87)	7.80 (4.62, 12.88)	
Thyroid disease history, %					0.6247
No	78.73 (73.45, 83.20)	80.02 (70.28, 87.15)	80.89 (72.82, 87.00)	76.31 (67.50, 83.32)	
Yes	21.27 (16.80, 26.55)	19.98 (12.85, 29.72)	19.11 (13.00, 27.18)	23.69 (16.68, 32.50)	
Liver disease history, %					0.2599
No	93.60 (89.53, 96.16)	97.29 (92.41, 99.06)	91.08 (80.78, 96.12)	92.95 (84.96, 96.85)	
Yes	6.40 (3.84, 10.47)	2.71 (0.94, 7.59)	8.92 (3.88, 19.22)	7.05 (3.15, 15.04)	
Cancer history, %					0.0076
No	80.11 (75.54, 84.01)	83.11 (73.39, 89.78)	87.04 (80.72, 91.51)	73.14 (66.16, 79.13)	
Yes	19.89 (15.99, 24.46)	16.89 (10.22, 26.61)	12.96 (8.49, 19.28)	26.86 (20.87, 33.84)	
Kidney failure history, %					<0.0001
No	94.57 (88.89, 97.43)	90.67 (81.45, 95.56)	91.21 (80.14, 96.39)	99.59 (98.40, 99.89)	
Yes	5.43 (2.57, 11.11)	9.33 (4.44, 18.55)	8.79 (3.61, 19.86)	0.41 (0.11, 1.60)	
Vital status, %					0.0175
Alive	78.60 (73.79, 82.74)	70.95 (63.63, 77.33)	79.89 (72.62, 85.62)	82.80 (75.47, 88.28)	
Deceased	21.40 (17.26, 26.21)	29.05 (22.67, 36.37)	20.11 (14.38, 27.38)	17.20 (11.72, 24.53)	

Family PIR, family poverty income ratio; BMI, body mass index; CVD, cardiovascular disease. For continuous variables: survey-weighted mean (95% CI), *p*-value was by survey-weighted linear regression, for categorical variables: survey-weighted percentage (95% CI), *p*-value was by survey-weighted chi-square test.

### 3.2 Kaplan–Meier survival analysis

To investigate the impact of OBS on prognosis in the OP population, we performed a Kaplan–Meier analysis. Individuals in the high OBS group showed substantially better survival outcomes than those in the low OBS group (*p* = 0.0032) (see Figure 2). Similar findings were obtained in the KM analysis for DOBS, where individuals in the high DOBS group showed higher survival probability (*p* = 0.001). However, no such trend was observed for LOBS (*p* = 0.54), which may be due to the limited range of LOBS values (ranging only between integers from 1 to 7), potentially requiring a larger sample size for further validation.

### 3.3 Relationship between OBS and all-cause mortality in the osteoporotic population

The association between OBS and all-cause mortality was analyzed during a median follow-up period of 6.75 years. Out of the 776 participants, 207 (26.7%) died. A weighted Cox proportional hazards regression model (see Table 2) was used to reveal the link between OBS and mortality. It was shown in Model 1 that all-cause mortality was negatively associated with OBS, indicating that higher OBS levels were linked to a lower mortality risk [HR = 0.95, 95% CI (0.93, 0.98), *p* = 0.0006]. Upon adjusting for multiple variables, an increase of one incremental unit in OBS corresponded to a 5% reduction in the risk of all-cause mortality [according to Model 2 analysis, HR = 0.95, 95% CI (0.93, 0.98), *p* = 0.0004], and this association remained after adjustment in Model 3, with a reduced risk of approximately 4% [HR = 0.96, 95% CI (0.94, 0.99), *p* = 0.0036]. Regression analysis based on OBS tertiles revealed that significantly lower risk of mortality in the high OBS group compared to the low OBS group: the hazard ratios for Models 1, 2, and 3 were found to be 0.54 [95% CI (0.34, 0.85), *p* = 0.0081], 0.59 [95% CI (0.39, 0.89), *p* = 0.0116], and 0.68 [95% CI (0.46, 0.99), *p* = 0.0422] respectively. Trend tests for all three models were significant: Model 1 (*p* for trend = 0.0082), Model 2 (*p* for trend = 0.0123), and Model 3 (*p* for trend = 0.0426). These findings suggest an independent negative association between OBS and all-cause mortality in OP individuals. Similar associations were observed for DOBS and LOBS in Model 3, after total adjustment, DOBS [HR = 0.97, 95% CI (0.95, 0.99), *p* = 0.0171] and LOBS [HR = 0.78, 95% CI (0.66, 0.92), *p* = 0.0030], indicating that an increase of one incremental unit in DOBS and LOBS was linked to 3 and 22% decrease in all-cause mortality risk, respectively. This suggests that while both antioxidant diets and lifestyles contribute to reducing all-cause mortality in OP patients, the relative effectiveness of an antioxidant lifestyle compared to an antioxidant diet requires further investigation due to the limited range of LOBS values and potential sample size constraints.

**Table 2:** Association between OBS/DOBS/LOBS and all-cause mortality in osteoporotic adults (weighted).

Characteristic	Model 1		Model 2		Model 3	
	HR (95% CI)	*p*-value	HR (95% CI)	*p*-value	HR (95% CI)	*p*-value
All-cause mortality						
OBS (continuous)	0.95 (0.93, 0.98)	0.0006	0.95 (0.93, 0.98)	0.0004	0.96 (0.94, 0.99)	0.0036
OBS (category)						
Low (5, 17)	Ref		Ref		Ref	
Medium (17, 23)	0.68 (0.46, 0.99)	0.0463	0.68 (0.46, 1.01)	0.0540	0.77 (0.49, 1.23)	0.2849
High (23, 36)	0.54 (0.34, 0.85)	0.0081	0.59 (0.39, 0.89)	0.0116	0.68 (0.46, 0.99)	0.0422
*p* for trend	0.0082		0.0123		0.0426	
DOBS (continuous)	0.95 (0.92, 0.98)	0.0013	0.96 (0.93, 0.98)	0.0017	0.97 (0.95, 0.99)	0.0171
DOBS (category)						
Low (3, 13)	Ref		Ref		Ref	
Medium (13, 19)	0.72 (0.47, 1.10)	0.1305	0.73 (0.49, 1.10)	0.1285	0.83 (0.55, 1.24)	0.3725
High (19, 29)	0.57 (0.35, 0.93)	0.0252	0.63 (0.41, 0.97)	0.0343	0.74 (0.50, 1.09)	0.1370
*p* for trend	0.0255		0.0356		0.1452	
LOBS (continuous)	0.89 (0.78, 1.01)	0.0825	0.84 (0.75, 0.94)	0.0026	0.78 (0.66, 0.92)	0.0030
LOBS (category)						
Low (1, 4)	Ref		Ref		Ref	
Medium (4, 5)	0.98 (0.56, 1.73)	0.9633	0.96 (0.54, 1.74)	0.9175	0.77 (0.45, 1.30)	0.3343
High (5, 7)	0.63 (0.44, 0.93)	0.0203	0.58 (0.39, 0.86)	0.0065	0.43 (0.25, 0.75)	0.0029
*p* for trend	0.0091		0.0011		0.0018	

Model 1, unadjusted; Model 2, adjusted for age group, gender, race; Model 3, adjusted for age group, gender, race, education level, family PIR, BMI, calcium, phosphorus, 25-OHD, hip fracture history, spinal fracture history, OP treatment history, hypertension history, dyslipidemia history, CVD history, diabetes history, thyroid disease history, liver disease history, cancer history, and kidney failure history.

### 3.4 Weighted restricted cubic spline analysis

**Figure 3:**
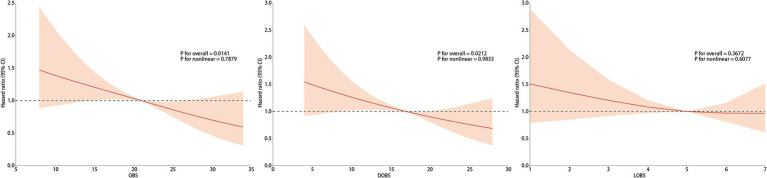
The association of OBS/DOBS/LOBS with all-cause mortality. Adjusted for age group, gender, race, education level, family PIR, BMI, calcium, phosphorus, 25-OHD, hip fracture history, spinal fracture history, OP treatment history, hypertension history, dyslipidemia history, CVD history, diabetes history, thyroid disease history, liver disease history, cancer history, and kidney failure history.

We applied weighted RCS to explore whether there was a linear association between OBS and all-cause mortality risk in patients with OP. Following adjustment for all confounding factors, the RCS analysis exhibited a notable linear inverse correlation with all-cause mortality among OP patients (*p* for nonlinear = 0.7879) (see Figure 3). Similarly, we observed a comparable linear negative link between DOBS and the all-cause mortality (*p* for nonlinear = 0.9833). However, LOBS did not reveal any significant linear association (*p* for overall = 0.3672), possibly due to the limited range of LOBS values (ranging from 1 to 7), which may be better treated as a categorical variable for analysis.

### 3.5 Subgroup analysis

**Figure 4:**
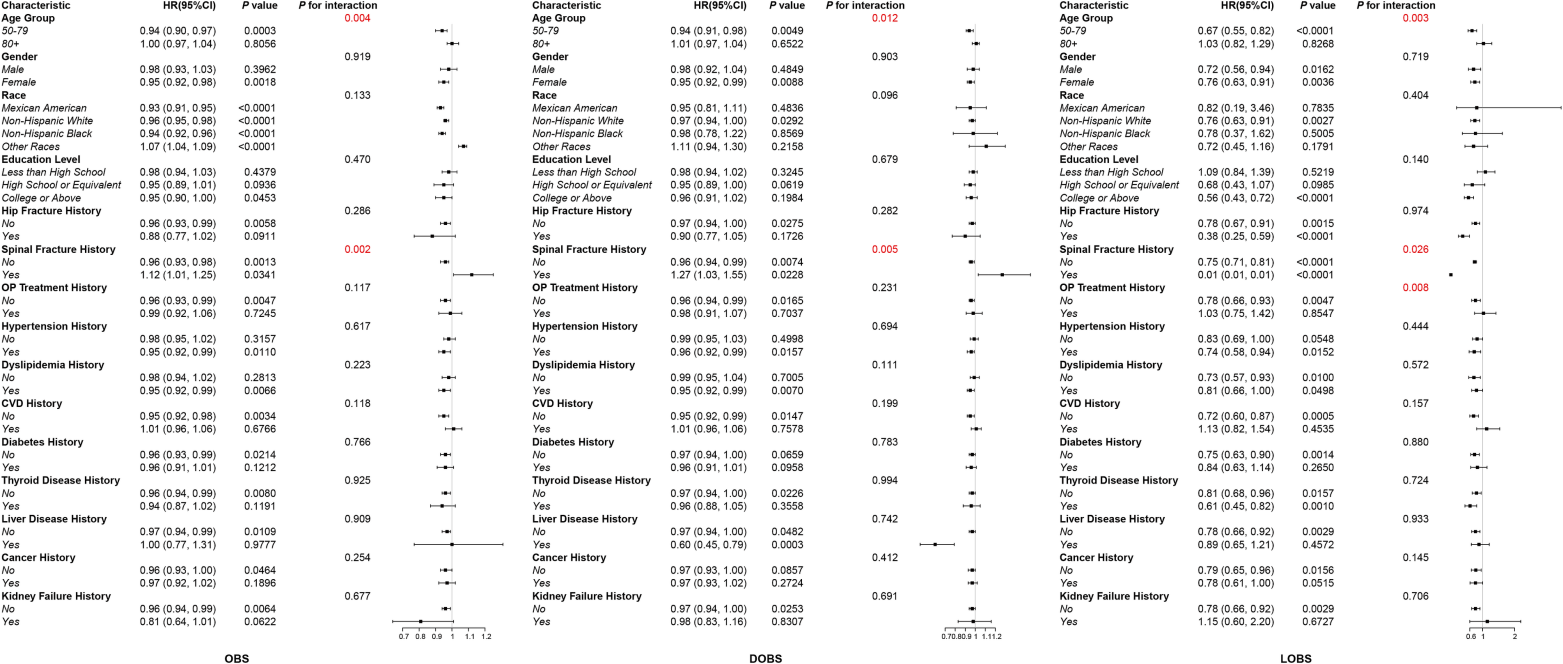
Subgroup analysis of the association between OBS/DOBS/LOBS and all-cause mortality in the osteoporotic population.

We conducted subgroup analyses and interaction tests to examine the potential link between OBS and all-cause mortality among different populations. At the same time, a weighted Cox proportional hazards model was utilized, adjusting for all covariates themselves, except for the subgroup variables themselves, to reveal potential interactions between OBS, DOBS, LOBS, and all-cause mortality. These relationships were examined across various subgroups defined by age and history of vertebral fracture. These relationships were examined across multiple subgroups, including those stratified by age and vertebral fracture history. Additionally, significant interactions between OBS and all-cause mortality were observed in the subgroup without OP treatment, while no significant interactions were found in the OP treatment subgroup (see Figure 4). The small sample size in the vertebral fracture history subgroup (only 24 participants) may have limited the ability to detect significant interactions, and larger studies are needed to confirm these findings. Among patients aged 50–79, OBS, DOBS, and LOBS were significantly negatively associated with all-cause mortality: OBS [HR = 0.94, 95% CI (0.90, 0.97), *p* = 0.0003], DOBS [HR = 0.94, 95% CI (0.91, 0.98), *p* = 0.0049], and LOBS [HR = 0.67, 95% CI (0.55, 0.82), *p* < 0.0001]. Interestingly, no significant associations were identified among patients aged 80 and older. In the OP treatment history subgroup, each one-unit increase in LOBS among patients without OP treatment was linked with a 22% decrease in all-cause mortality risk [HR = 0.78, 95% CI (0.66, 0.93), *p* = 0.0047], whereas no significant association was observed in patients with OP treatment history. Overall, the independent inverse link between OBS and all-cause mortality remained consistently evident across different subgroups within the OP population.

### 3.6 Sensitivity analysis

After removing individuals who passed away within the first 2 years of follow-up, the expanded interpretation of the sensitivity analysis results reveals that the study further confirms a notable inverse correlation between OBS and all-cause mortality risk [HR = 0.96, 95% CI (0.94, 0.99), *p* = 0.0106] (see Supplementary Table S3). Similar associations were observed for DOBS and LOBS with all-cause mortality: DOBS [HR = 0.97, 95% CI (0.94, 0.99), *p* = 0.0395] and LOBS [HR = 0.79, 95% CI (0.67, 0.93), *p* = 0.0057]. Therefore, the inverse association between oxidative balance score and mortality risk was most evident in the untreated group and remained consistent throughout the follow-up period.

## 4 Discussion

This research represents a pioneering effort to investigate the association between OBS and all-cause mortality among individuals diagnosed with OP. In 776 OP patients across five NHANES cycles (2005–2010, 2013–2014, 2017–2018), the Kaplan–Meier survival analysis of OBS tertiles, alongside the covariate-adjusted weighted Cox proportional hazards model, as well as subgroup and sensitivity analyses consistently demonstrated a strong association between elevated OBS levels and reduced all-cause mortality risk among patients diagnosed with OP, demonstrating an independent inverse correlation between OBS and all-cause mortality in this population. Incorporating antioxidant-rich diets and healthy lifestyles may help reduce oxidative stress and inflammation, thereby supporting bone health, reducing fracture risk, and contributing to the comprehensive prevention and management of osteoporosis.

OP is an age-related disease whose prevalence increases with an aging population. Fractures resulting from decreased BMD due to imbalanced bone mineral metabolism are significant contributors to increased all-cause mortality in the elderly (24). Excess ROS produced by oxidative stress impacts bone metabolism in various ways. First, ROS enhances the transcription of osteoclast-specific genes through the MAPK/NLRP3, NFATc/MMP9, and PERK/ATF4 pathways, promoting osteoclastogenesis and increasing osteoclast activity (25–27), which leads to increased bone resorption and is associated with RANKL activation. Furthermore, ROS can trigger osteoblast apoptosis through the PI3K/AKT/GSK3β signaling pathway (28), thereby reducing bone formation capacity. Wnt signaling is a key regulator of osteoblast differentiation, and the canonical Wnt signaling pathway involving β-catenin is activated by binding extracellular Wnt ligands (29). One study found that Wnt/β-catenin inhibits ROS, leading to the transdifferentiation of osteoblasts into chondrocytes and slowing bone mineralization (30). Moreover, FOXO-mediated oxidative stress can directly inhibit osteogenesis by suppressing Wnt signaling (31). Functional decoupling between osteoblasts and osteoclasts caused by these active oxygen species is a crucial reason for decreased bone mass and deteriorating bone health.

Moreover, a negative association has been observed between OBS and the prevalence of other diseases, including cardiovascular disease (32), diabetes (33), and metabolic syndrome (34), as well as cardiovascular and all-cause mortality risk in a population with diabetic and chronic kidney disease (CKD) populations (35, 36). A cohort study using NHANES datasets spanning 1999 to 2018 showed a close association between CKD occurrence and oxidative stress, suggesting that the reduction in mortality risk among CKD patients with higher OBS may be due to an enhanced antioxidant capacity, which provides protective effects on kidney health (36). Therefore, bone health deterioration and other complications caused by oxidative stress may jointly contribute to increased all-cause mortality in OP patients.

Our conclusions emphasize the significance of incorporating antioxidant diets and lifestyles into the prevention and comprehensive treatment strategies for OP patients. An antioxidant diet includes intake of plant-based nutrients, such as dietary fiber, polyphenols, carotenoids, and vitamins including vitamin A, riboflavin, vitamin B12, vitamin C, and vitamin E, along with regular physical activity to reduce oxidative stress. Conversely, excessive intake of pro-oxidants such as iron, as well as smoking, alcohol consumption, and obesity, may increase oxidative stress. As a key antioxidant, dietary fiber intake has been positively associated with lumbar spine BMD (37), while vitamin C (ascorbic acid) plays an essential role in neutralizing ROS and reducing oxidative stress (38). Increased dietary vitamin C intake has also been linked to a reduced risk of hip fracture, OP, and increased BMD, especially in the femoral neck and lumbar spine (39). Vitamin C has also been shown to stimulate osteoclast and osteoblast formation *in vitro*. Lycopene, an acyclic isomer of β-carotene (40), has been associated with reduced incidence of hip and nonvertebral fractures over a 17-year longitudinal study, highlighting its potential role in bone health (41). Increased lycopene levels may reduce oxidative stress and bone resorption markers, lowering the risk of OP. Nutrients in serum, such as selenium, have shown a positive correlation with calcaneal bone density (42), while serum ferritin levels were negatively correlated with bone density (43). The interactions between individual pro-oxidants and antioxidants within the body can be either antagonistic or synergistic, which is why we used OBS as a comprehensive measure of overall oxidative balance.

However, in the context of OP, analysis of the LOBS revealed no significant association with all-cause mortality. This finding was observed in the Kaplan–Meier survival analysis and weighted restricted cubic spline analysis. The reason may be due to the limited range of LOBS values (only between 1 and 7), as well as a small sample size, which may require larger sample sizes to investigate the association. In the weighted COX regression, significant associations were identified between OBS, DOBS, LOBS, and all-cause mortality among individuals diagnosed with OP. Therefore, it is necessary to increase antioxidant nutrient intake and adopt antioxidant lifestyles.

To gain deeper insights into the correlation between OBS and all-cause mortality in diverse populations, we performed subgroup analysis and interaction testing. These analyses yielded more profound insights into the impact of various factors on the association. Subgroup analysis showed significant interactions between OBS, DOBS, LOBS, and all-cause mortality with age and history of vertebral fracture. Among middle-aged and elderly individuals (<80 years), the inverse correlation between OBS, DOBS, LOBS, and all-cause mortality was notably pronounced. Furthermore, subgroup analysis showed that the inverse association between OBS and all-cause mortality was most evident in OP patients aged 50–79 years. This may be due to their relatively preserved physiological function, making them more susceptible to the harmful effects of oxidative stress, which is closely linked to chronic diseases such as cardiovascular disease and diabetes (44). In contrast, those aged ≥80 years may represent a survivor cohort with better intrinsic resilience to oxidative damage. Additionally, mortality in the very elderly is often influenced by factors like frailty and multimorbidity, which may not be strongly related to oxidative balance. Finally, the limited sample size and variability in OBS values in this group could reduce statistical power. These factors collectively may explain the age-specific differences observed. In those individuals aged 80 and above, considering that the contribution of specific diseases, such as cardiovascular disease and cancer, to overall mortality may differ among various subgroups, these variations may partly influence the observed association between OBS and all-cause mortality. After examining the data, we found that only 24 patients had a history of vertebral fractures, suggesting that this outcome may be driven by the small sample size, highlighting the need for more extensive studies to analyze this relationship.

This study has certain strengths. First, we rigorously selected and excluded participants from the NHANES database to explore the association between OBS and all-cause mortality among patients with OP. Additionally, a broad range of confounding variables was addressed, and diverse statistical approaches were applied to minimize bias and enhance result reliability. We systematically evaluated the association between OBS and mortality risk was examined by categorizing OBS, DOBS, and LOBS classifications. However, the study also has limitations. First, due to database limitations, we could not obtain more detailed information on OP patients, including imaging data and detailed treatment history. More detailed imaging assessments—such as measurements at additional skeletal sites (e.g., total hip), high-resolution peripheral quantitative computed tomography (HR-pQCT), and trabecular bone score (TBS)—were not analyzed, and these aspects need to be further strengthened. Second, the dietary components of OBS were derived from an average of two 24-h self-reported dietary recalls. This method may result in inaccuracies, as participants could misestimate their nutrient intake. Third, while ethnicity was adjusted for in the regression models, further subgroup analyses by ethnicity were not performed. Given the potential variations in osteoporosis risk factors across ethnic groups, this may limit the generalizability of our findings and warrants further investigation. Furthermore, although our study cohort was carefully selected and adjusted for major confounders, the relatively limited sample size and lack of external validation are acknowledged limitations. Future studies using larger, independent cohorts are warranted to validate these findings. Finally, the generalizability of our findings to individuals aged over 50 in other countries remains unclear and requires further investigation.

## 5 Conclusion

In this study, we observed that OBS is negatively associated with all-cause mortality in OP patients. Incorporating OBS into assessments could potentially be used in clinical practice to identify individuals at high risk of mortality, helping patients and the general population detect OP early and reduce the risk of all-cause mortality. Our results highlight the critical role of incorporating antioxidant diets and healthy lifestyles into the prevention and comprehensive treatment strategies for OP patients.

## Data Availability

Publicly available datasets were analyzed in this study. This data can be found at: https://www.cdc.gov/nchs/nhanes/index.htm.
